# A web-based cognitive behaviour therapy for chronic fatigue in type 1 diabetes (Dia-Fit): study protocol for a randomised controlled trial

**DOI:** 10.1186/s13063-015-0764-4

**Published:** 2015-06-06

**Authors:** Juliane Menting, Stephanie Nikolaus, Jan-Frederic Wiborg, Ellen Bazelmans, Martine M. Goedendorp, Arianne C. van Bon, Joop P. van den Bergh, Marc JTM. Mol, Cees J. Tack, Hans Knoop

**Affiliations:** Expert Center for Chronic Fatigue (ECCF), Radboud University Medical Center, P.O. Box 9101, 6500 HB Nijmegen, The Netherlands; Department of Medical Psychology, Radboud University Medical Center, Nijmegen, The Netherlands; Department of Health Psychology, University Medical Center Groningen, University of Groningen, Groningen, The Netherlands; Department of Internal Medicine, Rijnstate Ziekenhuis, Arnhem, The Netherlands; Department of Internal Medicine, VieCuri Medisch Centrum, Venlo, The Netherlands; Department of Internal Medicine, Canisius-Wilhelmina Ziekenhuis, Nijmegen, The Netherlands; Department of Internal Medicine, Radboud University Medical Center, Nijmegen, The Netherlands

**Keywords:** Chronic fatigue, Type 1 diabetes, Cognitive behaviour therapy, Blended-care, Internet, Study protocol, Randomised controlled trial

## Abstract

**Background:**

Fatigue is frequently reported by patients with type 1 diabetes mellitus. A recent study showed that 40 % of patients experienced severe fatigue that lasted for more than six months and was accompanied by substantial impairments in daily functioning. Currently, there is no effective treatment available for chronic fatigue in patients with type 1 diabetes. Cognitive behaviour therapy aimed at cognitions and behaviours that perpetuate fatigue is effective in reducing fatigue in other chronic diseases. Recent research showed that these cognitions and behaviours are also potential determinants of fatigue in type 1 diabetes. We designed Dia-Fit, a web-based cognitive behaviour therapy for severe and chronic fatigue in patients with type 1 diabetes. This patient-tailored intervention is aimed at reducing fatigue by changing cognitions and behaviours assumed to maintain fatigue. The efficacy of Dia-Fit will be investigated in this study.

**Methods/design:**

A randomised controlled trial will be conducted in 120 patients with type 1 diabetes who are chronically and severely fatigued. Patients will be randomised to a treatment or waiting list group. The treatment group will receive Dia-Fit, a blended care therapy consisting of up to eight internet modules and face-to-face sessions with a therapist during a five-month period. The treatment will be tailored to the fatigue-maintaining cognitions and behaviours that are relevant for the patient and are determined at baseline. The waiting list group will receive Dia-Fit after a waiting period of five months. The primary outcome measure is fatigue severity. Secondary outcome measures are functional impairment and glucose control determined by haemoglobin A_1c_ and blood glucose variability.

**Discussion:**

To our knowledge, this is the first study investigating the efficacy of a cognitive behavioural intervention for chronic fatigue in patients with type 1 diabetes.

**Trial registration:**

Dutch trial register NTR4312 (10 December 2013).

## Background

Diabetes mellitus is a highly prevalent health care problem: about 380 million adults are affected by diabetes worldwide and the number is expected to rise to 590 million adults by the year 2035 [1]. About 10 % of patients are diagnosed with type 1 diabetes mellitus (T1DM). T1DM is an autoimmune disorder, mostly diagnosed in childhood or adolescence, leading to beta cell destruction and an insulin secretion defect [2]. Patients need to inject insulin to control their blood glucose levels. T1DM is associated with medical complications both acute and long term, such as cardiovascular diseases, nephropathy, neuropathy and retinopathy [2]. The goal of diabetes treatment is to control blood glucose levels at a near normal level to delay or prevent medical complications and increase the quality of life of patients. Optimal diabetes control requires continuous diabetes self-management.

The proposed study described in this paper will focus on severely fatigued patients with T1DM. Fatigue is an often-reported symptom by patients with T1DM. In a cross-sectional study by our research group of 214 patients with T1DM, 40 % of patients suffered from severe fatigue lasting at least for six months [3]. Chronic fatigue was associated with more impairment in daily functioning, and fatigue was the most burdensome symptom of all assessed diabetes-related symptoms. [3]. Other studies that have investigated fatigue in T1DM patients provide limited information about the impact and chronicity of fatigue [4, 5]. However, fatigue is also a highly prevalent symptom in other chronic diseases. Fatigue was found to be a burdensome and invalidating symptom in patients with type 2 diabetes [6–8] and highly prevalent in patients with rheumatoid arthritis and neuromuscular disorders [9–11].

**Fig. 1:**
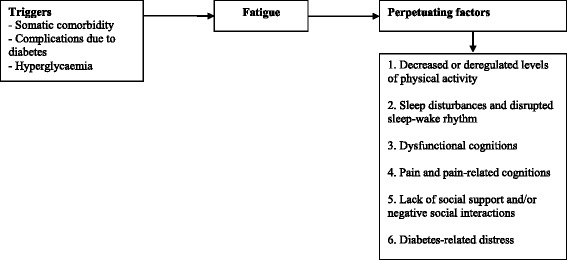
Model of perpetuating factors of fatigue in patients with T1DM

The etiology of severe fatigue in T1DM is not well understood. It seems obvious that physiological diabetes-related factors such as haemoglobin A1c (HbA_1c_) levels or variations in blood glucose levels are associated with fatigue. While fatigue is a classical presenting symptom of hyperglycaemia, in our cross-sectional study no relationship between glucose control (HbA_1c_ levels) and fatigue severity was found [3]. Also, parameters gathered during continuous glucose monitoring such as the blood glucose variability were unrelated to persistent fatigue. Other diabetes-related factors were correlated to fatigue: the number of complications due to diabetes, diabetes-related distress and diabetes specific self-efficacy. Also, there was a univariate relationship between fatigue and somatic comorbidity. In a multiple regression, several cognitive behavioural factors were found to be potential determinants of fatigue: disrupted sleep-wake patterns, low physical activity, catastrophising thoughts about fatigue and low self-efficacy with respect to fatigue and pain. These cognitive behavioural factors are also known to be determinants of fatigue in other chronic diseases [12–15]. Based on the available literature on diabetes and chronic fatigue in other chronic illnesses, we designed a cognitive behavioural model of fatigue in T1DM (Fig. 1). We assume that fatigue in T1DM is initially triggered by hyperglycaemia and/or diabetes-related factors such as the number of complications due to diabetes and/or somatic comorbidities. Once the fatigue has been triggered, other factors perpetuate it. We assume that these perpetuating factors are cognitive behavioural factors such as 1) a decreased or deregulated level of physical activity, 2) sleep disturbances and disrupted sleep-wake rhythm and 3) dysfunctional cognitions with respect to fatigue. Furthermore, we assume 4) pain and pain-related cognitions and 5) a lack of social support and/or negative social interactions to be perpetuating factors of fatigue in T1DM. All the aforementioned factors have been identified as determinants of fatigue in patients with T1DM and/or have repeatedly been found to be perpetuating factors of fatigue in other chronic illnesses [3, 12–15]. Finally, 6) diabetes-related distress is added to the model, because an association between fatigue and diabetes-related distress was found [3]. The six perpetuating factors can be addressed in cognitive behaviour therapy (CBT) for fatigue. Previous research has shown that CBT is effective in reducing fatigue in other chronic diseases and conditions [16–18].

To our knowledge, there are no studies that tested the efficacy of interventions specifically aimed at chronic fatigue in patients with T1DM. We propose that CBT aimed at the maintaining factors of fatigue will lead to a reduction of fatigue and associated disabilities. For this purpose, we developed Dia-Fit, a web-based cognitive behavioural intervention aimed at reducing fatigue. Dia-Fit is a blended-care therapy that consists of web-based modules supported by face-to-face sessions with a therapist. Blended care has the advantage of limiting the therapist time needed to deliver the intervention and reducing travel time, expenses and rigid appointments for patients. The primary objective of this study is to investigate the efficacy of Dia-Fit for chronic fatigue in patients with T1DM in a randomised controlled trial. The primary outcome measure is fatigue severity. Secondary outcome measures are the level of disabilities, HbA_1c_ and blood glucose variability. We will also investigate the long-term effects of Dia-Fit in a follow-up, six months after the intervention. If Dia-Fit leads to the expected improvement in fatigue severity compared to the waiting list, we will perform a mediation analysis to determine whether changes in the proposed fatigue maintaining factors mediate the effect of Dia-Fit on fatigue severity.

## Methods/design

### Study design

**Fig. 2:**
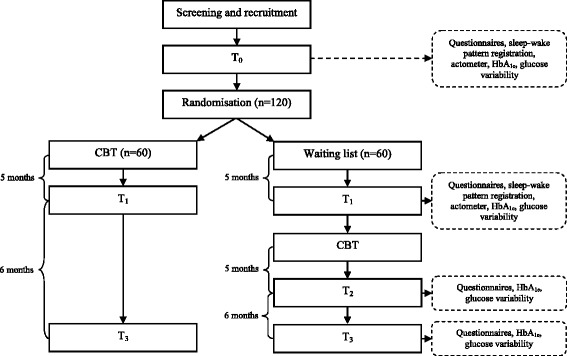
Flowchart of the trial design. T_0_ = baseline assessment; T_1_ = second assessment; T_2_ = post-treatment assessment for patients on the waiting list, T_3_ = follow-up assessment

The study is a randomised controlled trial (RCT) and will be conducted at the Expert Center for Chronic Fatigue (ECCF) of the Radboud University Medical Center. Patients who are eligible to participate will be randomly allocated to either the intervention group or a waiting list group. Patients allocated to the intervention group will directly receive the Dia-Fit intervention, while patients allocated to the waiting list group will receive the Dia-Fit intervention after a waiting period of five months. Assessments are planned before and after the intervention and the waiting list period. In both conditions, patients will be assessed again at a follow-up six months after receiving the treatment (Fig. 2).

### Recruitment process and study population

One hundred and twenty chronically fatigued patients with T1DM will be included. Patients will be recruited from the diabetes outpatient clinic of the Radboud University Medical Center and three general hospitals all located in the South East Netherlands. Patients will also be recruited through websites and social media. Patients of the diabetic outpatient clinics will be screened by their treating consultant for the sociodemographic and medical criteria of eligibility (criteria 1, 2 and 3 of the inclusion criteria and criteria 1 through 7 of the exclusion criteria). Inclusion criteria and exclusion criteria are listed in Table 1.

**Table 1:** Inclusion and exclusion criteria

	**Inclusion criteria**
(1)	Diagnosis of type 1 diabetes for at least 1 year
(2)	Between 18 and 70 years old
(3)	Able to read, speak and write Dutch
(4)	Severely fatigued operationalised as scoring ≥ 35 on the subscale fatigue severity of the CIS (Checklist Individual Strength)
(5)	Fatigued for at least 6 months
	**Exclusion criteria**
(1)	Moderate to severe renal failure operationalised as having a glomerular filtration rate (GFR) ≤ 45
(2)	Blindness or severe visual impairment
(3)	Medical history of congestive heart failure
(4)	Medical history of a stroke in the past five years
(5)	Body mass index (BMI) ≥ 40
(6)	Wheelchair-dependent
(7)	Other concurrent psychiatric or medical comorbidity that could explain the fatigue

Patients who are eligible will receive a letter from their consultant with information about the possibility of receiving treatment for persistent fatigue in the context of this research project. Attached to the letter, patients will receive a short screening questionnaire consisting of the Checklist Individual Strength (CIS) [19] and a question about the duration of the fatigue (criteria 4 and 5 of the inclusion criteria). Patients who are interested in participating can fill in the questionnaire and send it back, together with a written consent giving permission to the researcher (JM) to contact them. Patients who have a score of 35 or higher on the subscale fatigue severity of the CIS and who indicate that they are fatigued for six months or longer will receive further information about the study, both by telephone and in writing. Patients can decide to participate in the study within a period of two weeks from the time that the researcher contacted them. If patients are willing to participate, they will be asked to give written informed consent for participation in the study. They will then be invited for a baseline assessment at the ECCF. During baseline assessment, patients fill in the Beck Depression Inventory (BDI) [20] and the Symptom Check List (SCL90) [21]. Patients with a score of 4 or higher on the BDI and/or a total score of 164 or higher on the SCL90, which is higher than the mean and two standard deviations of healthy people from the general population [21], will be screened for the presence of a psychiatric disorder using the Mini-International Neuropsychiatric Interview (M.I.N.I.) [22]. Patients will be excluded if they meet the criteria of the M.I.N.I. for: depressive episode, suicidality, (hypo-)manic episode, panic disorder, obsessive-compulsive disorder, post-traumatic stress disorder, alcohol dependence and alcohol abuse, substance dependence and substance abuse, psychotic disorders, anorexia nervosa, bulimia nervosa and/or generalised anxiety disorder.

Patients who contact the researcher in response to information about the study in the media and are not treated in one of the four participating hospitals will first be screened for eligibility by a consultant of the diabetes outpatient clinic of the Radboud University Medical Center.

### Ethical approval

This study has been reviewed and approved by the Medical Ethical Committee of the Radboud University Medical Center (registration number 2013/165, NL43178.091.13). The study has also been approved and registered by the local ethical committees of the involved general hospitals: Rijnstate Ziekenhuis, Canisius-Wilhelmina Ziekenhuis and VieCuri Medisch Centrum. The study has been registered in the Dutch Trial Register (trial number NTR4312). All patients will receive verbal and written information about the study, and all patients must give written informed consent before randomisation and inclusion.

### Intervention

Dia-Fit consists of blended care, a combination of assignments, information and e-mail contact delivered via an internet portal and individual face-to-face sessions with a therapist. The total duration of the intervention is five months. During these months patients get online information and assignments, have fortnightly e-mail contact with their therapist and receive five to eight face-to-face sessions. The intervention is aimed at changing cognitions and behaviours thought to maintain fatigue; these are depicted in Fig. 1. Which cognitions and behaviours are relevant and applicable for the individual patient will be determined on the basis of cut-off scores on various questionnaires filled in at baseline assessment and the clinical interview by the therapist. For each fatigue-perpetuating factor a treatment module is developed. In this way the intervention can be tailored based on the applicable factors. Patients can follow from three up to eight of the following modules:

1. *Goals setting*. All patients receive this module. The module is the start of the Dia-Fit intervention and consists of psycho-education about fatigue in T1DM and the cognitive behavioural model of fatigue in T1DM. There will be a discussion about which modules are relevant for the patient. The patient will formulate goals of the therapy that, if attained, imply that a patient is no longer severely fatigued and no longer limited by fatigue in daily functioning.
2. *Regulation of the sleep-wake pattern*. At baseline, patients register bedtimes, times that they get up and the time slept during the day for two consecutive weeks. This module is indicated if patients score 60 or higher on the subscale sleep of the Sickness Impact Profile 8 (SIP8) [23] and/or if their bedtime and get-up time registration shows evidence of a disrupted sleep-wake pattern. In this module the importance of a regular sleep-wake cycle is discussed. Patients are asked to maintain fixed bedtimes and get-up times and to stop sleeping or lying down during the day.
3. *Formulating helpful fatigue-related beliefs*. This module addresses low self-efficacy with respect to fatigue, fatigue catastrophising and the tendency of patients to focus on fatigue. Dysfunctional beliefs will be reformulated and patients will practice applying helpful beliefs in their daily life. If patients score 19 or lower on the Self Efficacy Scale (SES) for fatigue [24] and/or 16 or higher on the Fatigue Catastrophising Scale (FCS) [25], they will receive this module. The tendency to focus on fatigue will be addressed if patients score 30 or higher on the Illness Management Questionnaire (IMQ) subscale focusing on symptoms [26]. Patients will learn how to shift their attention to other things instead of fatigue, such as activities or the environment. Patients will also be asked to stop talking about fatigue and to ask significant others to stop talking about fatigue.
4. *Activity regulation and increasing the level of activity.* This module is applicable for all patients and focuses on gradually increasing activity. The physical activity pattern of patients will be assessed with an actometer at baseline. An actometer is a small device which is worn at the ankle during two consecutive weeks [27]. On the basis of the scores of the actometer, each patient will be categorised in one of the two activity patterns: relatively active or low active. The physical activity of relatively active patients varies from day to day and is often characterised by ‘all or nothing’ behaviour. Relatively active patients first learn to divide their activities more evenly across the day and week and then increase their physical activity with a graded activity program. Patients can choose to increase their physical activity level either by walking or biking. They start walking or biking at least two times a day and increase their walking or biking time step by step. Patients with a low active pattern immediately start by increasing their physical activity. After patients have increased their physical activity, they apply the same principles to social or mental activities. Only patients who experience specific problems with social or mental activities will receive these elements of the module. Patients who do not need this specific step and believe that they are able to increase their level of activity will proceed with other modules and the realisation of their goals.
5. *Coping with pain*. This module focuses on dysfunctional cognitions regarding pain. It is assumed that catastrophising thoughts with respect to pain will make it difficult for patients to increase their activity level. Therefore, patients learn to use more helpful beliefs with respect to pain. Patients who score 55 or lower on the pain subscale of the SF36 [28] and/or 16 or higher on one of the two subscales, magnification and rumination, of the Pain Catastrophising Scale (PCS) [29] will receive this module.
6. *Optimalisation of social support and interactions.* T1DM patients with severe fatigue can experience problems in their interactions with significant others due to a lack of understanding or support. In this module patients learn how to improve their communication with significant others about fatigue. In exercises they learn how to communicate with others about fatigue and how to be more assertive. There is also an emphasis on having more realistic expectations with respect to the reaction of others. This module is indicated if patients score 50 or higher on the subscale discrepancy and/or score 14 or higher on the subscale negative interactions of the Sonderen Social Support Inventory (SSI) [30].
7. *Reducing diabetes-related distress*. T1DM is a chronic disease, and its management is demanding for patients. Patients can develop diabetes-related distress, for example, related to the fear of the development of medical complications, hypoglycaemia or deregulated blood glucose values. In this module patients concretise the elements of diabetes that they find stressful and learn how to better cope with these elements. The module is indicated if patients score 30 or higher on the Problem Areas in Diabetes (PAID) questionnaire [31].
8. *Step-by-step realisation of goals*. All patients end Dia-Fit with the realisation of goals. Patients realise the preset goals and evaluate the treatment effects. Patients who work less because of their fatigue will resume work in this module. Therapists will discuss with patients how to prevent relapse.

### Development of Dia-Fit and usability testing

The information and assignments provided on the Dia-Fit portal are developed by experts on chronic fatigue and type 1 diabetes. Usability testing was used to test the portal. Three patients with T1DM, recruited from the diabetes outpatient clinic of the Radboud University Medical Center, participated in the usability tests. They were interviewed about the usability of the portal and completed various tasks on the website in the presence of a researcher using the ‘think aloud’ technique. The intervention was improved on the basis of the findings of the usability test.

### Training, supervision and treatment integrity

All therapists are experienced cognitive behaviour therapists working at the ECCF. Therapists will be trained in delivering Dia-Fit. They will receive bi-weekly supervision from an experienced clinical psychologist (HK).

Treatment integrity will be determined by digitally recording all face-to-face sessions and saving all e-mail contacts of Dia-Fit. At the end of the study, 5 % of the sessions and the e-mail contacts will be randomly selected and evaluated to assess to what extent the Dia-Fit treatment was delivered according to protocol.

### Outcome measures

The primary outcome measure is fatigue severity measured with the fatigue severity subscale of the CIS [19]. The CIS subscale fatigue consists of eight items that are scored on a 7-point Likert scale from (1) ‘Yes, that is true’ to (7) ‘No, that is not true’. Scores range from 8 to 56 with higher scores indicating more severe fatigue. Severe fatigue is operationalised as scoring 35 and higher, which is higher than the mean plus two standard deviations of a healthy control group [32]. The CIS is a valid and reliable instrument that has been used before in patient groups with chronic diseases [12, 13].

Secondary outcome measures are limitations in daily functioning and diabetes control. Limitations in daily functioning will be measured with the total score on the Sickness Impact Profile 8 (SIP8) [23]. The SIP8 measures functional disability in eight different domains of functioning: sleep and rest, homemaking, mobility, social interactions, ambulation, leisure activities, alertness behaviour and work limitations. The eight subscale scores are added to provide one weighted score of disability (SIP8 total score). Higher scores indicate more disabilities. Diabetes control will be determined with two diabetes-specific clinical measurements: HbA_1c_ values and blood glucose variability. HbA_1c_ values are routinely assessed at the diabetes outpatient clinics every three months. Whenever possible, assessment of HbA_1c_ for the study and the routine assessment will be combined. Blood glucose variability will be derived from 7-point blood glucose profiles, measured for two consecutive days. The standard deviation of the mean glucose level is used as an indicator of blood glucose variability [33].

Questionnaires that will help decide which modules of the Dia-Fit intervention should be used are described in the section Intervention. Based on the model of fatigue in T1DM several fatigue- and diabetes-related cognitions and behaviours are assessed. Questionnaires and measurement points are listed in Table 2.

**Table 2:** Time points of all measures

	Measurements	T_0_	T_1_	T_2_	T_3_
**Main outcome measures**					
Fatigue severity	Checklist Individual Strength (CIS, subscale fatigue) [19]	X	X	X	X
**Secondary outcome measures**					
Limitations in daily functioning	Sickness Impact Profile 8 (SIP8, total score) [23]	X	X	X	X
Diabetes regulation	HbA_1c_; blood glucose variability [33]	X	X	X	X
**Indicators for modules**					
Sleep problems	Sickness Impact Profile 8 (SIP8, subscale sleep) [23]	X	X		
Dysfunctional cognitions with respect to fatigue	Self Efficacy Scale (SES) [24]	X	X		
	Fatigue Catastrophising Scale (FCS) [25]	X	X		
	Illness Management Questionnaire (IMQ) [26]	X	X		
Level of physical activity	Actometer [27]	X	X		
Pain severity and impact of pain	SF36 Questionnaire (subscale pain) [28]	X	X		
	Pain Catastrophising Scale (PCS) [29]	X	X		
Cognitions with respect to social support and social interactions	Sonderen Social Support Inventory (SSI) [30]	X	X		
Diabetes-related distress	Problem Areas in Diabetes (PAID) [31]	X	X		
**Other measures**					
Depression	Beck Depression Inventory (BDI) [20]	X	X		
Psychological distress	SCL90 [21]	X			
Cognitions regarding fatigue	Fatigue Quality List (FQL) [39]	X	X		
Cognitions with respect to activity	Tampa Scale for Kinesiophobia 2 (TSK2) [40]	X	X		
Affective quality of pain	McGill Pain Questionnaire (MPQ) [41]	X	X		
Causal attributions	CAL diabetes [42]	X	X		
Diabetes quality of life	Diabetes Quality of Life Brief Clinical Inventory (DQOL_BCI) [43]	X	X		
Cognitions regarding symptoms of chronic illness	Illness Cognition Questionnaire (ICQ) [44]	X	X		
	Cognitive and Behavioural Responses to Symptoms Questionnaire (CBRSQ) [45]	X	X		
Self-efficacy regarding diabetes self-care	Confidence in Diabetes Self-Care Scale (CIDS) [46]	X	X		
Physical activity	International Physical Activity Questionnaire (IPAQ) [47]	X	X		

T_0_ = baseline assessment; T_1_ = second assessment; T_2_ = post-treatment assessment for patients on the waiting list, T_3_ = follow-up assessment

### Assessments

The baseline assessment (T_0_) consists of two appointments at the ECCF. During both test sessions patients will complete questionnaires (Table 2). In the two weeks between the two sessions patients will wear an actometer to measure physical activity [27]. Patients will also record their symptoms and activities in a diary. In addition, blood glucose variability and HbA_1c_ will be assessed. After the baseline assessment patients will be randomised to either the Dia-Fit intervention group or the waiting list group. After five months the second assessment will be done (T_1_), consisting of the same measures as at baseline assessment. After T_1_ the waiting list group will start with Dia-Fit. The waiting list group will receive an extra assessment after therapy (T_2_). All patients will be assessed six months after finishing Dia-Fit (T_3_). T_2_ and T_3_ will consist of a limited number of measures (Table 2).

### Adverse events

Adverse events (AEs) will be assessed at T_1_. Patients will be asked to fill in a questionnaire regarding the development of new symptoms during the therapy or waiting period. All AEs that are spontaneously reported by patients or observed by the investigator will be recorded and reported to the ethical committee. The investigator will also record and report serious adverse events (SAEs) to the ethical committee. Previous research has shown that CBT for fatigue is a safe treatment [34].

### Treatment adherence

Treatment adherence will be determined in two ways. First, therapists will be asked to rate the degree of adherence to the Dia-Fit intervention by the patient on a scale of 0 to 10 at the end of the therapy. Second, patients will be asked to rate the degree to which they adhered to the different modules of the Dia-Fit treatment at the end of the therapy. Both scores will be correlated with the change score (pre-treatment versus post-treatment) on the primary outcome measure.

### Sample size

Sample size calculation was based on the guidelines of Borm and colleagues (2007) [35] and Van Breukelen (2006) [36] for analysis of covariance (ANCOVA) in randomised controlled trials. On the primary outcome parameter, the CIS fatigue severity, we assumed a clinically relevant difference in post-treatment scores of 6 between the Dia-Fit and the waiting list condition [37]. With a power of 0.90, a two-sided alpha of 0.05 and a standard deviation of 8.6, a minimum number of 45 patients would be needed per condition when using a *t*-test. According to Borm and colleagues (2007) this number of patients can be multiplied by a ‘design factor’ when ANCOVA is used. This factor is one minus the squared correlation coefficient between baseline and second assessment of fatigue severity. As we have no data on which to base this correction, we used a conservative estimate of 1 as a factor which corresponds to a relatively low correlation between baseline and second assessment of about r = .20 (1 - .20^2^ = .96 ≈ 1). Assuming a drop-out rate of 25 %, 60 patients per condition need to be randomised (n = 120 in total).

### Randomisation

Patients will be randomised in an equal ratio (1:1) to one of the two groups: 1) intervention group or 2) waiting list group. A computer randomisation program that is created by an independent statistical expert will be used for randomisation. Block randomisation is used with blocks of 6. Patients will be stratified into two groups: 1) patients recruited from hospitals and 2) patients recruited via media. A test assistant who is not involved in the study will do the random allocation in the presence of the patient after the baseline assessment. The researcher (JM) will also be present to plan appointments with the therapist and for the second assessment. The researcher is not blinded for treatment allocation. A researcher blinded for treatment allocation will do the statistical analysis.

### Statistical analysis

To test if there is a difference between the intervention group and the waiting list conditions on the primary outcome measure at second assessment (T_1_), ANCOVA will be used with the score on the second assessment as the dependent variable, the baseline score on the dependent measure as covariate, and condition as fixed factor [36]. Analysis of the data will be based on intention to treat. Missing values will be replaced with multiple imputation with fully conditional specification with at least five imputations. When statistically significant differences are found, a sensitivity analysis will be performed on the basis of different assumptions about the values of missing data. For the secondary outcome measures, limitations of daily functioning and diabetes control, the same analyses will be used. To determine if the expected positive result of CBT will be sustained at follow-up, scores at follow-up (T_3_) of patients treated with CBT directly or after the waiting period will be compared with the scores at post-treatment (T_1_ or T_2_) using paired *t*-tests.

Finally, we will test with multiple mediation which changes in the proposed fatigue maintaining cognitions and behaviours mediate the expected effect of the intervention on the primary outcome parameter of fatigue severity. The proposed mediators are factors that are thought to maintain fatigue severity. The mediation analysis will be conducted according to the approach of Preacher and Hayes [38]. Significance of the mediation effects will be determined using a non-parametric bootstrap approach which increases the power to detect significant effects even in small, non-normally distributed samples [38].

## Discussion

To our knowledge, this study is the first randomised controlled trial testing the efficacy of a web-based cognitive behavioural intervention for chronic fatigue in patients with T1DM. Chronic fatigue is highly prevalent in T1DM and is experienced by patients as one of the most disabling symptoms of the illness. An effective treatment focusing on fatigue in patients with T1D is not yet available.

The blended-care character of Dia-Fit, consisting of web-based information, assignments and e-mail contact supported by face-to-face sessions, is a promising approach for both patients and therapists. The tailored approach of Dia-Fit makes it possible to concentrate on fatigue maintaining factors that are relevant for each individual patient.

In conclusion, the results of the described study will provide information about the efficacy of CBT for severe fatigue in patients with T1DM and, one hopes, will contribute to the treatment of fatigue in T1DM.

## Trial status

Recruitment of the Dia-Fit study is ongoing. The recruitment started in January 2014 and is expected to end in February 2016.

## Abbreviations

AE: adverse event
ANCOVA: analysis of covariance
CBT: cognitive behaviour therapy
CIS: Checklist Individual Strength
ECCF: Expert Center for Chronic Fatigue
HbA_1c_: haemoglobin A_1c_
RCT: randomised controlled trial
SAE: serious adverse event
SIP: Sickness Impact Profile
T1DM: type 1 diabetes mellitus
